# Side Effects of mRNA-Based and Viral Vector-Based COVID-19 Vaccines among German Healthcare Workers

**DOI:** 10.3390/biology10080752

**Published:** 2021-08-05

**Authors:** Miloslav Klugar, Abanoub Riad, Mohamed Mekhemar, Jonas Conrad, Mayte Buchbender, Hans-Peter Howaldt, Sameh Attia

**Affiliations:** 1 Czech National Centre for Evidence-Based Healthcare and Knowledge Translation (Cochrane Czech Republic, Czech EBHC: JBI Centre of Excellence, Masaryk University GRADE Centre), Institute of Biostatistics and Analyses, Faculty of Medicine, Masaryk University, Kamenice 5, 625 00 Brno, Czech Republic; klugar@med.muni.cz (M.K.); abanoub.riad@med.muni.cz (A.R.); 2 Institute of Health Information and Statistics of the Czech Republic, Palackého náměstí 4, 128 01 Prague, Czech Republic; 3 Department of Public Health, Faculty of Medicine, Masaryk University, Kamenice 5, 625 00 Brno, Czech Republic; 4 Clinic for Conservative Dentistry and Periodontology, School of Dental Medicine, Kiel University, Arnold Heller Str. 3, Haus B, 24105 Kiel, Germany; mekhemar@konspar.uni-kiel.de (M.M.); conrad@konspar.uni-kiel.de (J.C.); 5 Department of Oral and Maxillofacial Surgery, University of Erlangen-Nuremberg, Glückstraße 11, 91054 Erlangen, Germany; mayte.buchbender@uk-erlangen.de; 6 Department of Oral and Maxillofacial Surgery, Justus-Liebig-University, Klinikstrasse 33, 35392 Giessen, Germany; HP.Howaldt@uniklinikum-giessen.de

**Keywords:** adverse effects, BTN162 vaccine, ChAdOx1 COVID-19 vaccine, cross-sectional studies, COVID-19 vaccines, drug-related side effects and adverse reactions, Germany, health personnel, mRNA-1273 vaccine, prevalence

## Abstract

**Simple Summary:**

The main way to overcome the COVID-19 pandemic is mass vaccination of the public. However, the public’s vaccine hesitancy toward the available vaccines is a big challenge in the fighting against the coronavirus spreading. We aimed in this study to report for the first time the short-term side effects following mRNA-based (Pfizer-BioNTech and Moderna) and viral vector-based (AstraZeneca) COVID-19 vaccines among German healthcare workers. A survey-based study was conducted through an online validated questionnaire. Overall, 88.1% of the German healthcare workers included in this study reported at least one side effect following the COVID-19 vaccination. The mRNA-based vaccines were associated with a higher prevalence of local side effects (e.g., injection site pain), while the viral vector-based vaccine was associated with a higher prevalence of systemic side effects (e.g., headache/fatigue). The vast majority (84.9%) of side effects resolved within 1–3 days after vaccination, which are promising results from a safety point of view for both types of vaccines. This study is one of the few studies that aims to enhance our emerging knowledge about the risk factors of COVID-19 vaccines side effects by inquiring and analyzing the self-reported side effects across various demographic and medical parameters.

**Abstract:**

Background: the increasing number of COVID-19 vaccines available to the public may trigger hesitancy or selectivity towards vaccination. This study aimed to evaluate the post-vaccination side effects of the different vaccines approved in Germany; Methods: a cross-sectional survey-based study was carried out using an online questionnaire validated and tested for a priori reliability. The questionnaire inquired about demographic data, medical and COVID-19-related anamneses, and local, systemic, oral, and skin-related side effects following COVID-19 vaccination; Results: out of the 599 participating healthcare workers, 72.3% were females, and 79.1% received mRNA-based vaccines, while 20.9% received a viral vector-based vaccine. 88.1% of the participants reported at least one side effect. Injection site pain (75.6%) was the most common local side effect, and headache/fatigue (53.6%), muscle pain (33.2%), malaise (25%), chills (23%), and joint pain (21.2%) were the most common systemic side effects. The vast majority (84.9%) of side effects resolved within 1–3 days post-vaccination; Conclusions: the mRNA-based vaccines were associated with a higher prevalence of local side effects (78.3% vs. 70.4%; *Sig.* = 0.064), while the viral vector-based vaccine was associated with a higher prevalence of systemic side effects (87.2% vs. 61%; *Sig.* < 0.001). Females and the younger age group were associated with an increased risk of side effects either after mRNA-based or viral vector-based vaccines. The gender- and age-based differences warrant further rigorous investigation and standardized methodology.

## 1. Introduction

Since the spreading of the COVID-19 pandemic, its influence has become evident worldwide in all disciplines and sectors, e.g., social, financial, and health sectors, etc. We were clearly unprepared for such a circumstance, which was entirely unfamiliar to the current generation. Over time, it was clear that overcoming this pandemic can only be done through mass vaccination [1].

Vaccination against infectious diseases such as SARS-CoV-2 is the most cost-effective public health intervention. In addition to individual immunization, the achievement of collective protection (so-called community immunity) for the majority of vaccine-preventable infections is also crucial to protect vulnerable groups in the population who, for various reasons, cannot be vaccinated [2].

The currently available vaccines against SARS-CoV-2 are manufactured by one of the following technologies: (a) mRNA-based vaccines, (b) viral vector-based vaccines, (c) protein subunit vaccines, and (d) whole virus or inactivated virus vaccines [3]. Heretofore, the European Medicines Agency (EMA) has approved vaccines that only belong to the first two technologies (mRNA-based and viral vector-based vaccines), which aim to produce spike protein-specific antibodies [4]. The mRNA-based technology is relatively novel in vaccine industry, and it employs molecular templates of messenger RNA (mRNA) to deliver the genetic information to produce the spike (S) glycoprotein antigen, not to deliver the antigen itself [5]. The viral vector-based vaccines against SARS-CoV-2 use a non-replicating harmless version of adenovirus as a vehicle to deliver the genetic code of the S glycoprotein antigen, thus eliciting the targeted immune response [6].

In Germany, a country with a population of around 84 million, 3,729,682 COVID-19 cases with 91,007 deaths were reported by 1 July 2021 [7,8]. To date, four COVID-19 vaccines had been approved in Germany; Pfizer-BioNTech (mRNA-based vaccine) approved since 21 December 2020, Moderna (mRNA-based vaccine) approved since 6 January 2021, AstraZeneca-Oxford (viral vector-based vaccine) approved since 29 January 2021, and Janssen (viral vector-based vaccine) approved since 11 March 2021 [9].

On 1 July 2021, 926,463 vaccine doses were administered in Germany, leading to 31.487.487 people (37.9% of the total population) being fully vaccinated and 46,249,449 people (55.6%) receiving at least one vaccine dose [10]. The German government imported -to date- 57,619,463 doses of Pfizer-BioNTech, 13,869,863 doses of AstraZeneca-Oxford, 7,641,280 doses of Moderna, and 2,893,697 doses of Janssen [10,11]. The Germany’s vaccination strategy prioritized healthcare workers to receive the vaccine, especially those who worked in the frontlines and treated COVID-19 patients [11,12].

In a recent cross-sectional study, Bauernfeind et al., 2021 investigated the opinions of the healthcare workers in Germany about COVID-19 vaccination [13]. This study revealed that 59.5% of the surveyed subjects were willing to get vaccinated, 21.4% were hesitant, and 18.7% were against getting vaccinated, thus bolding the need for innovative strategies to tackle vaccine hesitancy and resistance drivers among German healthcare workers [13]. Aversion to side effects had been widely recognized as one of the key drivers of vaccine hesitancy that requires transparent and independent safety evidence of the vaccines, especially the novel ones [14].

The overarching aim of this study was to investigate the short-term side effects following COVID-19 vaccines reported by German healthcare workers. The primary objective was to estimate the prevalence of the side effects of both mRNA-based and viral vector-based vaccines. The secondary objectives were (a) to evaluate the demographic and medical risk factors of the COVID-19 vaccines side effects; and (b) to compare between the side effects frequency and intensity of mRNA-based versus viral vector-based vaccines.

## 2. Materials and Methods

### 2.1. Design

A cross-sectional survey-based study had been designed as a post-marketing (phase IV) trial between February and April 2021 to evaluate the self-reported side effects of COVID-19 vaccines among healthcare workers in the Federal Republic of Germany. The study utilized a self-administered questionnaire (SAQ) developed and delivered online through KoboToolbox (Harvard Humanitarian Initiative, Cambridge, MA, USA) for data collection [15]. The study protocol was registered a priori at the U.S. National Library of Medicine (NLM) under the identifier NCT04706156 [16,17]. The study was entirely conducted and reported according to Strengthening the Reporting of Observational Studies in Epidemiology (STROBE) guidelines for cross-sectional studies [18].

### 2.2. Participants

The eligible participants of this study were healthcare workers who had been vaccinated among the priority groups recommended by the Standing Committee on Vaccination (STIKO) of Robert Koch Institute (Berlin, Germany) during the first quarter of 2021 [11]. The healthcare workers who received the Pfizer-BioNTech vaccine (BTN162 vaccine), Moderna vaccine (mRNA-1273 vaccine), and the AstraZeneca-Oxford vaccine (ChAdOx1 COVID-19 vaccine) were included in this study regardless of the number of doses they had received by the time of filling the questionnaire. While the Janssen vaccine (Ad26.COV2.S vaccine) was authorized by the EMA since 11 March 2021 it was not yet deployed on large scale among the German population when the study was carried out, and it was not even offered to the priority group of healthcare workers. Therefore, we used the AstraZeneca-Oxford vaccine as a representative for the viral vector technology.

A non-random sampling technique was used in recruiting the participants, as printed posters within the vaccination center (Kiel, Schleswig-Holstein, Germany) were used to promote the study among the target participants. Additionally, a snow-balling technique was used to invite participants from other recruitment locations (Bayern, Bavaria, Germany) and (Giessen, Hessen, Germany).

Participation in this study was entirely voluntary, and the participants received no financial compensation in order to minimize the risks of response bias and performance bias. The participants were allowed to withdraw from the study at any moment until data submission without the need to justify their decision.

**Figure 1:**
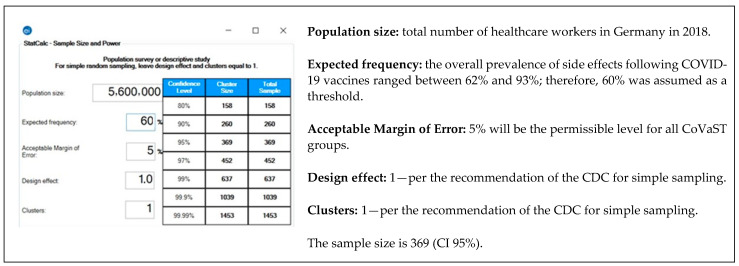
Sample size of healthcare workers (HCWs) in Germany—Epi-Info ^TM^ version 7.2.4. [19,23].

The sample size was calculated using Epi Info ^TM^ version 7.2.4 (CDC. Atlanta, GA. 2020). The formula of population survey studies was used to achieve 5% of error margin and 95% confidence level [19]. The expected frequency (outcome probability) is assumed to be 60% as the prevalence of side effects following COVID-19 vaccines ranged between 62% to 93% in our previous studies [20,21,22] (Figure 1).

### 2.3. Instrument

The SAQ used in this study consisted of 28 multiple-choice items, which were adapted from the safety reports of phase III trials published by the Centers for Disease Control and Prevention (CDC, Atlanta, GA, USA) for the mRNA-based vaccines, and the European Medicines Agency (EMA, Amsterdam, The Netherlands) for the viral vector-based vaccine [24,25,26].

The validation and reliability testing process was described in detail previously elsewhere [20]. The content validity was assessed by an experts’ panel, and the test re-test reliability of the suggested SAQ yielded a mean Cohen’s kappa coefficient of 0.89 ± 0.13 (0.54–1), thus indicating substantial reliability [20].

The items of the SAQ were stratified into four main categories: (a) demographic information including gender, age, profession, work experience, and federal state; (b) medical anamneses including chronic disease and medical treatments; (c) COVID-19-related anamnesis including previous infection, exposure, type of vaccine, number of doses; and (d) the local, systemic, oral, and skin-related side effects of COVID-19 vaccines [20,21,22].

### 2.4. Ethics

The study was fully reviewed and approved by the Ethics Committee of the Faculty of Medicine at the Justus Liebig University of Giessen (Ref. 55/20). The study data was controlled and processed by Masaryk University (MUNI) in full compliance with the General Data Protection Regulation (GDPR) [27]. A digital informed consent was obtained from each participant before participation in the study, and no identifying personal information was collected that may facilitate the retrospective identification of the participants.

### 2.5. Analysis

All statistical tests were executed by the Statistical Package for the Social Sciences (SPSS) version 27.0 (SPSS Inc., Chicago, IL, USA, 2020) [28]. The normality of data was evaluated using the Shapiro–Wilk test with a significance level (*Sig.*) of 0.05. Primarily, descriptive statistics was used to present and summarize the categorical variables like gender, profession, work experience, region, medical anamneses, COVID-19-related anamneses, and individual side effects by frequency (n) and percentage (%). The continuous variables were age, number of chronic illnesses and medical treatments, and number of side effects either locally, systemically, orally, or dermatologically by mean (*μ*) and standard deviation (*SD*).

Chi-squared test (*χ*^2^), Fisher’s exact test, Mann–Whitney (*U*) test, Kruskal–Wallis (*H*) test were used to estimate the association between demographic and medical risk factors of COVID-19 vaccines and evaluate the differences between mRNA-based and viral vector-based COVID-19 vaccines. Binary logistic regression for the side effects occurrence either locally, systemically, or generally was used to evaluate the proposed demographic and medical risk factors. All inferential tests were carried out assuming a confidence level (CI) of 95% and a significance value of 0.05.

## 3. Results

### 3.1. Demographic Characteristics

A total of 599 participants completed the SAQ properly; therefore, they were included in the final analysis. While 386 participants received Pfizer-BioNTech and 88 received Moderna (mRNA-based vaccine; *n* = 474), 125 received AstraZeneca-Oxford (viral vector-based vaccine; *n* = 125). The included participants received their first dose of the vaccines between 27 December 2020 and 30 March 2021.

The optimal sample size was reached for the total number of participants and for participants with Pfizer-BioNTech vaccine. The median age of the participants was 39 years old; therefore, it was used in the downstream analyses as a cut-off for age-dependent comparisons. Out of the 474 mRNA-based vaccine recipients, 73.6% were females, 50.4% were ≤ 39 years-old, 51.3% were nurses, 40.9% had > 20 years of work experience, and 75.7% were from Schleswig-Holstein. Out of the 125 viral vector-based vaccine recipients, 67.2% were females, 50.4% were ≤ 39 years-old, 40.8% were nurses, 37.6% had 1–5 years of work experience, and 70.4% were from Schleswig-Holstein (Table 1).

### 3.2. Medical Anamneses

Overall, 29.5% and 24.8% of mRNA-based vaccine and viral vector-based vaccine recipients reported at least one non-communicable disease, respectively. The most common chronic illness among mRNA-based vaccine recipients was thyroid disease (7.6%), followed by chronic hypertension (6.1%), and asthma (6.1%). Similarly, thyroid disease (8.8%) was the most common illness among viral vector-based vaccine recipients, followed by chronic hypertension (5.6%). The only significant difference between mRNA-based vaccine recipients and viral vector-based vaccine recipients (*χ*^2^ = 4.115 and 7.146; *Sig.* = 0.043 and 0.030) was in terms of asthma (6.1% vs. 1.6%) and chronic obstructive pulmonary disease (0.2% vs. 2.4%).

While 35.4% of mRNA-based vaccine recipients were taking medications regularly, only 24% of viral vector-based vaccine recipients were taking them (*χ*^2^ = 5.853; *Sig.* = 0.016). Antihypertensive drugs (12.9%) were the most common among mRNA-based vaccine recipients, followed by thyroid hormone supplements (8.9%), contraceptives (5.3%), immunosuppressive drugs (3.8%), and antidepressants (3.6%). In the viral vector-based vaccine group, thyroid hormone supplements (8.8%) were the most common medication, followed by antihypertensive drugs (5.6%), antidepressants (5.6%), antidepressants (5.6%), and contraceptives (3.2%) (Table 2).

### 3.3. COVID-19-Related Anamneses

However, the vast majority of mRNA-based vaccine group (90.3%) received two doses, the vast majority of viral vector-based vaccine group (99.2%) received only one dose by the time they filled in the SAQ (*χ*^2^ = 389.771; *Sig.* < 0.001). Only four recipients of mRNA vaccine and two recipients of viral vector-based vaccine were previously infected by SARS-CoV-2. In total, 175 (29.2%) participants reported being exposed to COVID-19 patients during the last months (Table 3).

### 3.4. Local and Systemic Side Effects

All local side effects, related to the injection site, were more prevalent in the mRNA-based vaccine group than the viral vector-based vaccine group. A total of 78.3% and 70.4% of mRNA-based vaccine and viral vector-based vaccine recipients reported at least one local side effect (*χ*^2^ = 3.421; *Sig.* = 0.064), respectively. Overall, injection site pain (75.6%) was the most prevalent local side effect, followed by injection site swelling (18%) and injection site redness (10.4%). Injection site pain (77.4% vs. 68.8%, respectively) was significantly more common in the mRNA-based vaccine group compared to the viral vector-based vaccine group (*χ*^2^ = 3.993; *Sig.* = 0.046).

On the contrary, all systemic side effects were more prevalent in the viral vector-based vaccine group than the mRNA-based vaccine group. A total of 87.2% and 61% of viral vector-based vaccine and mRNA-based vaccine recipients reported at least one systemic side effect (*χ*^2^ = 30.522; *Sig.* < 0.001), respectively. Overall, the most common systemic side effect was headache/fatigue (53.6%), followed by muscle pain (33.2%), malaise (25%), chills (23%), and joint pain (21.2%). The differences between the viral vector-based vaccine group and mRNA-based vaccine group were statistically significant (*χ*^2^ = 97.782, 106.419, 27.506, 27.292, 63.907, 16.161 and 47.501; *Sig.* < 0.001, < 0.001, < 0.001, < 0.001, < 0.001, < 0.001 and < 0.001, respectively) in terms of fever (48% vs. 9.9%), chills (57.6% vs. 13.9%), headache/fatigue (74.4% vs. 48.1%), muscle pain (52.8% vs. 28.1%), joint pain (47.2% vs. 14.3%), nausea (20.8% vs. 8.2%), and malaise (48.8% vs. 18.8%).

In general, 86.3% and 80% of the side effects reported by mRNA-based vaccine and viral vector-based recipients remained 1–3 days. The severe side effects that required medical attention were reported only by two (0.4%) mRNA-based vaccine recipients and four (3.2%) viral vector-based vaccine recipients (*Sig.* = 0.019; 2-S Fisher’s exact test) (Table 4).

### 3.5. Oral and Skin-Related Side Effects

A total of 106 (17.7%) participants reported experiencing at least one oral side effect, with 12.4% and 37.6% of mRNA-based vaccine and viral vector-based vaccine recipients being affected (*χ*^2^ = 42.967; *Sig.* < 0.001), respectively.

The most prevalent oral side effect was vesicles (6.3%), followed by bleeding gingiva (4.3%), halitosis (3.7%), oral paranesthesia (2.2%), swollen mucosa (2.2%), and ulcers (2%). Taste disturbance (6.4% vs. 0.8%), vesicles (12.8% vs. 4.6%), halitosis (10.4% vs. 1.9%), bleeding gingiva (12% vs. 2.3%), and xerostomia (2.4% vs. 0%) were significantly more common among viral vector-based vaccine recipients.

More than three-fourths (75.6%) of oral side effects emerged within the first week after vaccination. The most common site for ulcers, vesicles and blisters were labial/buccal mucosa (43.2%), followed by lips (29.5%) and tongue (27.3%). Tongue (57.1%) and labial/buccal mucosa (57.1%) were the common sites for white/red plaque (Table 5).

A total of 21 (3.5%) participants reported experiencing at least one skin-related side effect, with 3% and 5.6% of mRNA-based vaccine and viral vector-based vaccine recipients being affected (*Sig.* = 0.171; 2-S Fisher’s exact test), respectively.

The most prevalent skin-related side effect was rash (2.8%), followed by urticaria (0.7%), and angioedema (0.7%). The most common affected sites were face (57.1%), followed by upper limb (38.1%), and lower limb (19%) (Table 6).

### 3.6. COVID-19 Vaccines Side Effects by Gender

The local side effects were almost equally distributed between female (78.2%) and male (77.9%) participants who received mRNA-based vaccines. On the other hand, the females who received the viral vector-based vaccine had a significantly higher prevalence of local side effects (*χ*^2^ = 5.989; *Sig.* = 0.014) compared to their male counterparts, 77.4% vs. 56.1, respectively.

Similarly, the difference between female and male participants in terms of systemic side effects was not statistically significant in the case of mRNA-based vaccines (*χ*^2^ = 1.868; *Sig.* = 0.172), but it was statistically significant in the case of the viral vector-based vaccine (*χ*^2^ = 4.578; *Sig.* = 0.032).

**Figure 2:**
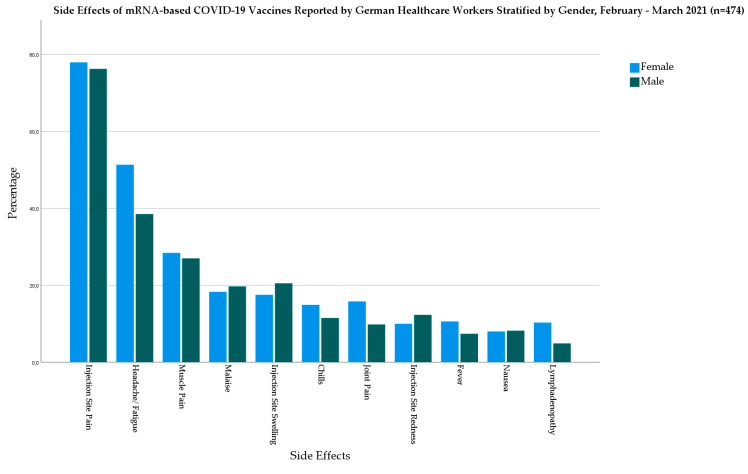
Side Effects of mRNA-based COVID-19 Vaccines Reported by German Healthcare Workers Stratified by Gender, February–March 2021 (*n* = 474).

In the mRNA-based vaccine group, females had higher prevalence of fever (10.6% vs. 7.4%), chills (14.9% vs. 11.5), headache/fatigue (51.3% vs. 38.5%), muscle pain (28.4% vs. 27%), joint pain (15.8% vs. 9.8%), and lymphadenopathy (10.3% vs. 4.9%) than males (Figure 2).

**Figure 3:**
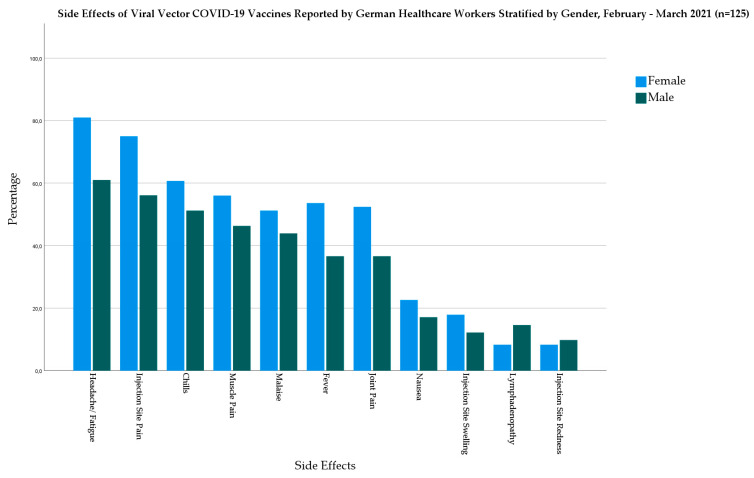
Side Effects of Viral Vector-based COVID-19 Vaccines Reported by German Healthcare Workers Stratified by Gender, February–March 2021 (*n* = 125).

In the viral vector-based vaccine group, females had higher prevalence of fever (53.6% vs. 36.6%), chills (60.7% vs. 51.2%), headache/fatigue (81% vs. 61%), muscle pain (56% vs. 46.3%), joint pain (52.4% vs. 36.6%), nausea (22.6% vs. 17.1%), and malaise (51.2% vs. 43.9%) (Figure 3).

The severe side effects were exclusively reported by females (n = 2) in the mRNA-based vaccine, and in the viral vector-based vaccine, the female:male ratio was 3:1. Oral side effects affected males slightly more than females in the mRNA-based vaccine group (14.8% vs. 11.7%) and the viral vector-based vaccine group (39% vs. 36.9%). In the mRNA-based vaccine group, all the skin-related side effects were reported by females (Table 7).

### 3.7. COVID-19 Vaccines Side Effects by Age

The local side effects were almost equally distributed between the younger age group (≤ 39 years-old) and the older age group (≤ 39 years-old) participants who received mRNA-based vaccines (78.7% vs. 77.9%, respectively) and the viral vector-based vaccine (73% vs. 67.7%, respectively).

The difference between the younger age group and older age group participants in terms of systemic side effects was statistically significant in case of mRNA-based vaccines (*χ*^2^ = 8.281; *Sig.* = 0.004), but it was not statistically significant in case of the viral vector-based vaccine (*χ*^2^ = 1.075; *Sig.* = 0.300). While the younger age group was more affected by systemic side effects following mRNA-based vaccines (67.4% vs. 54.5%), they were less affected by systemic side effects following viral vector-based vaccine (84.1% vs. 90.3%) compared to the older age group.

**Figure 4:**
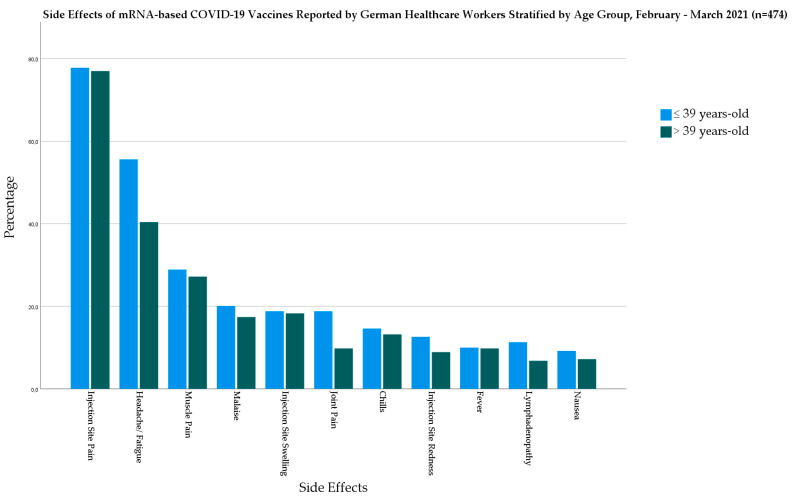
Side Effects of mRNA-based COVID-19 Vaccines Reported by German Healthcare Workers Stratified by Age Group, February–March 2021 (*n* = 474).

In the mRNA-based vaccine group, the younger age group had a significantly higher level of headache/fatigue (*χ*^2^ = 11; *Sig.* = 0.001) and joint pain (*χ*^2^ = 7.882; *Sig.* = 0.005) than the older age group (Figure 4).

**Figure 5:**
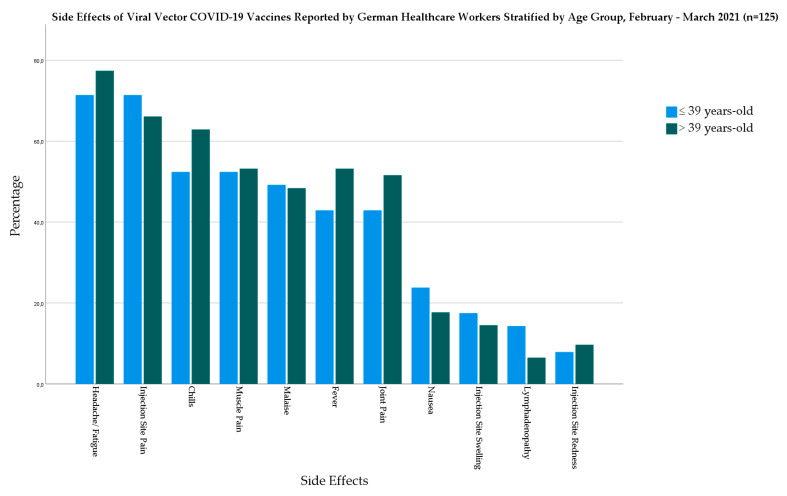
Side Effects of Viral Vector COVID-19 Vaccines Reported by German Healthcare Workers Stratified by Age Group, February–March 2021 (*n* = 125).

In the viral vector-based vaccine group, the older age group had a higher prevalence of most of the investigated systemic side effects but without statistical significance. The largest difference between the age groups was in the case of chills (10.5%), followed by fever (10.3%), and joint pain (8.7%) (Figure 5).

The severe side effects were exclusively reported by the younger age group (*n* = 2) in the mRNA-based vaccine, and in the viral vector-based vaccine, the older age group:younger age group ratio was 3:1. Oral side effects affected the younger age group slightly more than the older age group in the mRNA-based vaccine group (13.8% vs. 11.1%) and the viral vector-based vaccine group (41.3% vs. 33.9%) (Table 8).

### 3.8. Risk Factors of COVID-19 Vaccine Side Effects

On performing binary logistic regression for the demographic and medical risk factors, female gender (only for viral vector vaccine), the younger age group, chronic illnesses (only for viral vector vaccine), and medications were associated with an increased odds ratio (OR) of COVID-19 vaccine side effects, however not statistically significant. The previous infection statistically significantly increased OR of side effects for both vaccine types 21.310 for mRNA-based vaccine and 7.721 for viral vector-based vaccine.

Female participants were 3.429 times (CI 95%: 0.910–12.912) more likely to experience side effects after viral vector-based vaccine than their male counterparts. The participants with chronic illnesses and taking medications were 2.173 times (CI 95%: 0.571–8.270) and 3.6 times (CI 95%: 0.965–13.428) more likely to experience side effects after the viral vector-based vaccine (Table 9).

In the mRNA-based vaccine group, females had OR of 1.338 times (CI 95%: 0.881–2.032) for systemic side effects and OR of 1.021 times (CI 95%: 0.621–1.679) for local side effects. The younger age group had OR of 1.725 times (CI 95%: 1.188–2.505) for systemic side effects and OR of 1.047 times (CI 95%: 0.677–1.621) for local side effects. The chronic illnesses increased the OR for systemic side effects slightly; similarly, the medications increased the OR for local side effects slightly (Table 10).

In the viral vector-based vaccine group, females had OR of 3.094 times (CI 95%: 1.061–9.022) for systemic side effects and OR of 2.677 times (CI 95%: 1.202–5.965) for local side effects. The participants with chronic illnesses had OR of 2.016 times (CI 95%: 0.667–7.094) for systemic side effects and OR of 1.182 times (CI 95%: 0.492–2.836) for local side effects. The participants taking medications regularly had OR of 2.125 times (CI 95%: 0.701–6.441) for systemic side effects and OR of 2.739 times (CI 95%: 1.162–6.455) for local side effects (Table 11).

## 4. Discussion

This post-marketing study demonstrated that 88.1% of the surveyed German healthcare workers reported at least one side effect after receiving COVID-19 vaccines; 87.1% following mRNA-based vaccines and 92% following the viral vector-based vaccine.

The cross-vaccine comparison of our sample data revealed that mRNA-based vaccines were associated with more frequent local side effects (78.3% vs. 70.4%); while the viral vector-based vaccine was associated with more frequent systemic side effects (87.2% vs. 61%). The largest post-marketing study to date of COVID-19 vaccines analyzed the side effects of BNT162b2 (mRNA-based vaccine) and ChAdOx1 nCoV-19 (viral vector-based vaccine) reported by UK inhabitants using the COVID-19 Symptom Study app (ZOE Global, London, UK) [29,30]. Our results are consistent with the findings of this UK study; as the mRNA-based vaccine was associated with a higher prevalence of local side effects (71.7% vs. 58.7%), while the viral vector-based vaccine was associated with a higher prevalence of systemic side effects (33.7% vs. 20%) among the British population [30]. Similarly, Mathioudakis et al., 2021 found that local side effects were more frequent in the mRNA-based vaccine group, while the systemic side effects were more frequent in the viral vector-based vaccine group [31]. Abu-Hammad et al., 2021 found that the mRNA-based vaccine was significantly associated with local side effects, while the viral vector-based vaccine was associated with systemic side effects among Jordanian healthcare workers [32]. On the contrary, Alhazmi et al., 2021 found no significant difference in terms of local side effects among vaccinated individuals in Saudi Arabia; however, the viral vector-based vaccine was still significantly associated with an increased risk of systemic side effects [33].

While the vast majority of governments rely on more than one vaccine in their mass-vaccination strategies, there is a lack of evidence on the comparative side effects of different COVID-19 vaccines [34,35]. The current evidence is limited by a series of constraints, including unequal sample size across the study groups, lack of normal distribution of demographic and medical risk factors across the study groups, and lack of attention to the onset of each side effect [30,31,32,33,36]. Vaccine selectivity can be defined as “the discriminatory attitudes towards certain types of vaccines based on their target contagion or manufacturing technology that yields heterogeneous acceptance levels of recommended vaccines”. Understandably, public health researchers might be diverted from comparing the side effects of different COVID-19 vaccines to avoid triggering vaccine selectivity through misinterpretation of their results. Nevertheless, the infodemic related to vaccine safety has been pragmatically targeting specific types of COVID-19 vaccines to increase vaccine hesitancy or trigger public selectivity against these vaccines [37,38]. Therefore, it is imperative to expand in the cross-vaccine comparison research while sticking to the highest standards of epidemiologic methodology to synthesize rigorous evidence that should fairly inform the public and individuals’ decision of vaccination.

The younger age (≤39 years-old) group were 1.122 times (CI 95%: 0.683–1.842) more likely to experience side effects compared to the older age (>39 years-old) group. The age-related differences in our sample were only statistically significant in case of systemic side effects that affected the mRNA-based vaccine recipients (67.4% vs. 54.5%; *Sig.* = 0.004). Nonetheless, the older age group had more systemic side effects following viral vector-based vaccine than the younger group. Menni et al., 2021 found that the British individuals aged 55 years or below had significantly higher prevalence of side effects following both mRNA-based and viral vector-based vaccines [30]. Similarly, Jordanian healthcare workers (≤45 years-old) and UAE inhabitants (≤49 years-old) reported more side effects following COVID-19 vaccination [32,39]. Mazur et al., 2021 found that the incidence of post-vaccination oral side effects decreases in the older age groups among the included European healthcare workers [36]. In Czech Republic and Turkey, the younger healthcare workers reported a higher incidence of short-term side effects following BNT162b2 and CoronaVac vaccines, respectively, compared to their older colleagues [20,22]. In contrast to the supposition that “the younger you are, the more likely to experience side effects”, El-Shitany et al., 2021 found that the Saudi individuals aged 60 years or above had a significantly higher level of local side effects, especially injection site pain (80.8% vs. 68.6%; *Sig.* = 0.0056) compared to their younger counterparts who received BNT162b2 vaccine [40].

The increased odds of side effects among young adults can be explained by the fact that these side effects are a by-product of the exuberant production of type I interferon (IFN-I) that occurs to initiate an effective immune response to the invading pathogen [41]. The generation of IFN-I in females and younger adults was found to be more potent [41,42]. The pre-marketing (phase III) and post-marketing (phase IV) studies of COVID-19 vaccines used heterogonous cut-off points for the age-related analyses of vaccines reactogenicity and side effects. While the phase III trials used the retirement age cut-offs, e.g., 55 years-old and 65 years-old, some phase IV studies were inclined to use the median age of their own surveyed samples in order to compare the prevalence of post-vaccination side effects [20,30,40]. Both approaches are unequivocally effective in evaluating and communicating age-related differences; nevertheless, harmonizing vaccine safety reports, especially those from independent institutions, became a methodological must. Therefore, the findings of this study call for developing consensus guidelines for reporting COVID-19 vaccine side effects and effectiveness.

Female participants in our study had OR of 0.833 (CI 95%: 0.441–1.573) and 3.429 (CI 95%: 0.910–12.912) to experience side effects following mRNA-based vaccines and viral vector-based vaccine, respectively, thus indicating that viral vector-based vaccines impact females more significantly, while mRNA-based vaccines had no statistically significant lower OR in females. We can hypothesize that if an optimal sample size was reached for the viral vector-based vaccine this result would be more likely also statistically significant. Interestingly, all side effects were more common among female recipients of viral vector-based vaccine except injection site redness and lymphadenopathy, which were slightly more common among male recipients. Alghamdi et al., 2021 found within a viral vector-based vaccine recipients sample that the prevalence of post-vaccination side effects was significantly higher in females than males [43]. They also found that the onset of side effects occurrence is faster in females, and also the intensity of side effects and the rate of pain killer consumption were significantly higher in females [43]. The female predominance was also reported in mRNA-based vaccines and inactivated virus vaccines [20,22,40].

The more vigorous immune response and the lower pain threshold of females are suggested hypotheses to explain the gender-based differences in self-reported COVID-19 vaccines side effects [44,45]. The selection bias and the information bias may also play a key role in the emergence of gender-based differences; therefore, gender-adjusted analyses are indispensable in studying the self-reported outcomes of COVID-19 vaccines. Females were consistently associated with an increased risk of side effects following viral vaccines, including influenza, measles–mumps–rubella combination vaccine (MMR), attenuated Japanese encephalitis, and attenuated Dengue vaccines [44,46]. Future research should focus on the gender-based differences of COVID-19 vaccines side effects.

Injection site pain was the most prevalent local side effect in our sample (75.6%), followed by injection swelling (18%) and injection site redness (10.4%). The same order was found in both vaccines, the UK, Jordan, Saudi Arabia, and the Czech Republic [20,30,32,40]. In general, all the systemic side effects were significantly more common among viral vector-based vaccine recipients than mRNA-based vaccine recipients.

The most common systemic side effect among our mRNA-based vaccine recipients was headache/fatigue (48.1%), followed by muscle pain (28.1%), malaise (18.8%), joint pain (14.3%), chills (13.9%), and fever (9.9%). In the safety report of the Centers for Disease Control and Prevention (CDC) for BNT162b2 vaccine, headache/fatigue (44.1%) was the most common systemic side effect reported by the phase III volunteers, followed by muscle pain (25.4%), chills (19.7%), joint pain (15%), and fever (7.9%) [24]. The CDC report of mRNA-1273 vaccine revealed that headache/fatigue (47.8%) was the most common systemic side effect, followed by muscle pain (40%), joint pain (29%), chills (25.3%), and fever (7.9%) [25]. The prevalence of mRNA-based vaccine systemic side effects in our sample was lower than what was reported in the phase III trials [24,25]. The similar finding was reported by the UK study were the side effects prevalence among the app user being significantly lower than the manufacturers’ reports [30]. Contrarily, Riad et al., 2021 found that the post-marketing prevalence of BNT162b2 side effects among Czech healthcare workers was higher than what was reported by the manufacturer [20].

The most common systemic side effect among our viral vector-based vaccine recipients was headache/fatigue (74.4%), followed by chills (57.6%), muscle pain (52.8%), malaise (48.8%), fever (48%), and joint pain (47.2%). In the safety report of the European Medicines Agency (EMA) for ChAdOx1 nCoV-19 vaccine, headache (52.7%), fatigue (53%), malaise (44.4%), muscle pain (43.9%), fever (41.1%), chills (32.2%), and joint pain (26.6%) [26]. The increased prevalence of side effects among our sample can be explained by the fact that the frequency of ChAdOx1 nCoV-19 side effects decreases after the second dose while our sample participants received only the first dose [26].

Manfredi et al., 2021 reported a case of a middle-aged female recipient of BNT162b2 vaccine who presented to their clinic with diffuse ulcerative lesions on the floor of the mouth associated with angular cheilitis and erythema of the tongue after two days of her first dose [47]. Poly(ethylene glycol) (PEG) as a constituent component of the mRNA-based vaccine was suspected to trigger these oral side effects [47,48]. Azzi et al., 2021 reported another case of a middle-aged female recipient of ChAdOx1 nCoV-19 with diffuse, erythematous and swollen red lesions on her buccal mucosa, tongue, gingiva and palate [49]. Heterozygous Factor V Leiden mutation was suspected to be the trigger of the thromboembolic events that were experienced by the patient, in addition to being a predisposing factor for the oral mucositis episode that followed the COVID-19 vaccination [49,50]. Oral mucosal lesions were increasingly reported in COVID-19 patients in the last months; therefore, the oral side effects of COVID-19 vaccines may mimic the COVID-19-associated oral symptoms [51,52,53,54,55,56,57,58,59].

In our sample, 17.7% of the participants reported at least one oral side effect, with mucosal lesions being the most commonly reported side effects, followed by bleeding gingiva (4.3%), halitosis (3.7%), and oral paranesthesia (2.2%), and taste disturbance (2%). It is worthy of mentioning that oral paranesthesia and taste disturbance were not solicited in our original questionnaire, even though the participants reported it voluntarily in the additional comment boxes. Therefore, we suggest that the actual prevalence of oral paranesthesia and taste disturbance can be higher than what we report in this study. The participants referred to oral paranesthesia by keywords, such as tongue tingling, mouth-tingling, and pins and needles sensation, e.g., while they referred to the taste disturbance by keywords like metallic taste, taste change, salty taste, and unpleasant taste, etc.

A large registry-based study by McMahon et al., 2021 revealed that mRNA-based vaccines were associated with a myriad of skin-related side effects that mimicked the SARS-CoV-2 infection [60]. In our sample 17 (2.8%) participants reported experiencing rash after vaccination, 4 (0.7%) reported urticaria, and 4 (0.7%) reported angioedema. The most common location of skin-related side effects was face (57.1%), followed by upper limb (38.1%), and lower limb (19%). However, there is a paucity of focus on the less common side effects, such as skin-related side effects by the phase IV trials of COVID-19 vaccines, and there is an emerging number of individual case reports and case series for recently vaccinated individuals with skin-related side effects [61].

### 4.1. Strengths

This is the first study to evaluate the side effects of COVID-19 vaccines among the German population to the best of the authors’ knowledge. This study is one of the few studies that aims to enhance our emerging knowledge about the risk factors of COVID-19 vaccines side effects by inquiring and analyzing the self-reported side effects across various demographic and medical parameters [20,21,22,30,31,40,62].

Another contribution of this study is its focus on the less reported side effects, e.g., the oral and skin-related ones, which were not solicited in the manufacturers’ reports of the phase III trials. However, the less common side effects being mild to moderate, may act as a trigger for a vaccine hesitancy or vaccine resistance position by the vaccinated individuals or their household and acquaintances because they were not clearly explained a priori. The optimal sample size was reached for a total number of participants and for participants with Pfizer-BioNTech vaccine.

### 4.2. Limitations

The first limitation of this study is that the vast majority of viral vector-based vaccine recipients received only the first dose by the time they filled in the questionnaire; therefore, it was not possible to compare between the first and the second dose side effects. However, this corresponds with the public health strategy to extend the period between the first and second dose, especially among viral vector-based vaccines. Another limitation is because we did not ask about the timing of each inquired side effect, whether it was after the first dose, second dose, or both doses. The software used for data collection, KoBoToolbox, does not enable the researchers to learn the number of form visitors (potential respondents) which is the denominator of the response rate equation; therefore, we could not calculate the response rate in our study.

While this study, like typical post-marketing (phase IV) trials, relies primarily on the self-reported outcomes of the respondents, it had targeted healthcare workers as they are deemed to retain substantial levels of health literacy. The optimal sample size was not reached for the number of participants with Moderna and AstraZeneca-Oxford vaccines. This limitation should be seen in the context of the national vaccination strategy that limited the number of healthcare workers who received Moderna and AstraZeneca-Oxford vaccines.

### 4.3. Implications

This study implies that future research of COVID-19 vaccine safety should focus on cross-vaccine comparison as it can deliver timely and critical messages to the public. The findings of this study strengthen the call for consensus guidelines for reporting the independent studies of COVID-19 reactogenicity and side effects to overcome the growing heterogeneity among the reports of different research groups worldwide.

The age and gender-related differences of local and systemic side effects prevalence and incidence warrant further investigation. The less common side effects, e.g., oral and skin-related side effects, should be widely tracked by independent vaccine safety studies. The onset of each side effect and its duration should be precisely inquired about in future vaccine safety studies.

The findings of this study serve as independent evidence on the safety of COVID-19 vaccines that should encourage the public to take informed decisions for getting vaccinated, as the non-serious side effects we found were of limited duration (1–3 days),mainly related to the injection site and not interfering with the daily routine.

## 5. Conclusions

Overall, 88.1% of the German healthcare workers included in this study reported at least one side effect following the COVID-19 vaccination. The mRNA-based vaccines were associated with a higher prevalence of local side effects, while the viral vector-based vaccine was associated with a higher prevalence of systemic side effects. Females and the younger age group were associated with an increased risk of side effects either after mRNA-based or viral vector-based vaccines. The gender- and age-based differences warrant further rigorous investigation and standardized methodology. Injection site pain was the most common local side effect, and headache/fatigue, muscle pain, malaise, chills, and joint pain were the most common systemic side effects. More than one-sixth of the participants reported at least one oral side effect, including mucosal lesions, oral paresthesia, and taste disturbance. The vast majority (84.9%) of side effects resolved within 1–3 days after vaccination, which is a positive message to the public about the short-term safety of vaccines.

## Figures and Tables

**Table 1:** Demographic Characteristics of Participating German Healthcare Workers, February–March 2021 (*n* = 599).

Variable	Outcome	mRNA Vaccine	Viral Vector Vaccine	Total
Gender	Female	349 (73.6%)	84 (67.2%)	433 (72.3%)
	Male	122 (25.7%)	41 (32.8%)	163 (27.2%)
	Non-binary	2 (0.4%)	0 (0%)	2 (0.3%)
	Prefer not to say	1 (0.2%)	0 (0%)	1 (0.2%)
Age	≤39 years-old	239 (50.4%)	63 (50.4%)	302 (50.4%)
	>39 years-old	235 (49.6%)	62 (49.6%)	297 (49.6%)
Profession	Physician	60 (12.7%)	35 (28%)	95 (15.9%)
	Dentist	62 (13.1%)	20 (16%)	82 (13.7%)
	Nurse	243 (51.3%)	51 (40.8%)	294 (49.1%)
	Pharmacist	4 (0.8%)	3 (2.4%)	7 (1.2%)
	Lab Worker	16 (3.4%)	2 (1.6%)	18 (3%)
	Psychologist	15 (3.2%)	0 (0%)	15 (2.5%)
	Dietitian	3 (0.6%)	2 (1.6%)	5 (0.8%)
	Physiotherapist	8 (1.7%)	0 (0%)	8 (1.3%)
	Paramedic	7 (1.5%)	8 (6.4%)	15 (2.5%)
	Midwife	3 (0.6%)	0 (0%)	3 (0.5%)
	Other	53 (11.2%)	4 (3.2%)	57 (9.5%)
Work Experience	1–5 years	146 (30.8%)	47 (37.6%)	193 (32.2%)
	6–10 years	55 (11.6%)	16 (12.8%)	71 (11.9%)
	11–20 years	79 (16.7%)	20 (16%)	99 (16.5%)
	>20 years	194 (40.9%)	42 (33.6%)	236 (39.4%)
State	Schleswig-Holstein	359 (75.7%)	88 (70.4%)	447 (74.6%)
	Bavaria	77 (16.2%)	23 (18.4%)	100 (16.7%)
	Hessen	27 (5.7%)	13 (10.4%)	40 (6.7%)
	Nordrhein-Westfalen	4 (0.8%)	1 (0.8%)	5 (0.8%)
	Berlin	2 (0.4%)	0 (0%)	2 (0.3%)
	Other	5 (1.1%)	0 (0%)	5 (0.8%)

**Table 2:** Medical Anamneses of Participating German Healthcare Workers, February–March 2021 (*n* = 599).

Variable	Outcome	mRNA Vaccine	Viral Vector Vaccine	Total	Sig.
Noncommunicable Diseases (NCDs)	Allergy	3 (0.6%)	1 (0.8%)	4 (0.7%)	1.000 *
	Asthma	29 (6.1%)	2 (1.6%)	31 (5.2%)	0.043
	Blood Disease	3 (0.6%)	1 (0.8%)	4 (0.7%)	1.000 *
	Bone Disease	5 (1.1%)	1 (0.8%)	6 (1%)	1.000 *
	Bowel Disease	10 (2.1%)	3 (2.4%)	13 (2.2%)	0.740 *
	Cancer	4 (0.8%)	3 (2.4%)	7 (1.2%)	0.162 *
	Cardiac Disease	10 (2.1%)	1 (0.8%)	11 (1.8%)	0.474 *
	Chronic Hypertension	29 (6.1%)	7 (5.6%)	36 (6%)	0.828
	COPD	1 (0.2%)	3 (2.4%)	4 (0.7%)	0.030
	Dermatologic Disorder	5 (1.1%)	1 (0.8%)	6 (1%)	1.000 *
	Diabetes Mellitus	9 (1.9%)	0 (0%)	9 (1.5%)	0.216 *
	Hepatic Disease	2 (0.4%)	0 (0%)	2 (0.3%)	1.000 *
	Neurologic Disorder	6 (1.3%)	3 (2.4%)	9 (1.5%)	0.404 *
	Ophthalmic Disease	1 (0.2%)	0 (0%)	1 (0.2%)	1.000 *
	Otolaryngologic Disease	1 (0.2%)	0 (0%)	1 (0.2%)	1.000 *
	Renal Disease	0 (0%)	1 (0.8%)	1 (0.2%)	0.209 *
	Rheumatoid Arthritis	13 (2.7%)	3 (2.4%)	16 (2.7%)	1.000 *
	Thyroid Disease	36 (7.6%)	11 (8.8%)	47 (7.8%)	0.656
	Intensity (*μ* ± SD)	0.30 ± 0.46	0.25 ± 0.43	0.29 ± 0.54	0.354
	Total	140 (29.5%)	31 (24.8%)	171 (28.5%)	0.30
Medications	Antiarrhythmics	2 (0.4%)	0 (0%)	2 (0.3%)	1.000 *
	Antiasthma	3 (0.6%)	1 (0.8%)	4 (0.7%)	1.000 *
	Antibiotics	3 (0.6%)	1 (0.8%)	4 (0.7%)	1.000 *
	Anticoagulants	9 (1.9%)	0 (0%)	9 (1.5%)	0.216 *
	Antidepressants	17 (3.6%)	7 (5.6%)	24 (4%)	0.280 *
	Antidiabetics	3 (0.6%)	2 (1.6%)	5 (0.8%)	0.280 *
	Antiepileptics	4 (0.8%)	2 (1.6%)	6 (1%)	0.610 *
	Antihistamine	8 (1.7%)	0 (0%)	8 (1.3%)	0.215 *
	Antihypertensive	61 (12.9%)	7 (5.6%)	68 (11.4%)	0.023
	Cholesterol-lowering	3 (0.6%)	0 (0%)	3 (0.5%)	1.000 *
	Contraceptives	25 (5.3%)	4 (3.2%)	29 (4.8%)	0.336
	Eye Drops	1 (0.2%)	0 (0%)	1 (0.2%)	1.000 *
	Immunosuppressives	18 (3.8%)	2 (1.6%)	20 (3.3%)	0.277 *
	Narcotic Analgesics	1 (0.2%)	3 (2.4%)	4 (0.7%)	0.030 *
	Thyroid Hormone	42 (8.9%)	11 (8.8%)	53 (8.8%)	0.983
	Intensity (*μ* ± SD)	0.42 ± 0.63	0.32 ± 0.63	0.40 ± 0.63	0.031
	Total	168 (35.4%)	30 (24%)	198 (33.1%)	0.016

Chi-squared test, Fisher’s exact test (*) and Mann–Whitney test were used with a significance level of <0.05.

**Table 3:** COVID-19-related Anamneses of Particpating German Healthcare Workers, February–March 2021 (*n* = 599).

Variable	Outcome	mRNA Vaccine	Viral Vector Vaccine	Total	Sig.
Doses	One	46 (9.7%)	124 (99.2%)	170 (28.4%)	<0.001
	Two	428 (90.3%)	1 (0.8%)	429 (71.6%)	
Infection	Yes	4 (0.8%)	2 (1.6%)	6 (1%)	0.610 *
Exposure	Yes	143 (30.2%)	32 (25.6%)	175 (29.2%)	0.318

Chi-squared test, Fisher’s exact test (*) and Mann–Whitney test were used with a significance level of <0.05.

**Table 4:** COVID-19 Vaccines General Side Effects of Participating German Healthcare Workers, February–March 2021 (*n* = 599).

Variable	Outcome	mRNA Vaccine	Viral Vector Vaccine	Total	Sig.
Local SE	Injection Site Pain	367 (77.4%)	86 (68.8%)	453 (75.6%)	0.046
	Injection Site Swelling	88 (18.6%)	20 (16%)	108 (18%)	0.507
	Injection Site Redness	51 (10.8%)	11 (8.8%)	62 (10.4%)	0.522
	Intensity (*μ* ± SD)	1.07 ± 0.80	0.94 ± 0.81	1.04 ± 0.81	0.069
	Total	371 (78.3%)	88 (70.4%)	459 (76.6%)	0.064
Systemic SE	Fever	47 (9.9%)	60 (48%)	107 (17.9%)	<0.001
	Chills	66 (13.9%)	72 (57.6%)	138 (23%)	<0.001
	Headache/Fatigue	228 (48.1%)	93 (74.4%)	321 (53.6%)	<0.001
	Muscle Pain	133 (28.1%)	66 (52.8%)	199 (33.2%)	<0.001
	Joint Pain	68 (14.3%)	59 (47.2%)	127 (21.2%)	<0.001
	Nausea	39 (8.2%)	26 (20.8%)	65 (10.9%)	<0.001
	Malaise	89 (18.8%)	61 (48.8%)	150 (25%)	<0.001
	Lymphadenopathy	43 (9.1%)	13 (10.4%)	56 (9.3%)	0.650
	Intensity (*μ* ± SD)	1.50 ± 1.75	3.60 ± 2.25	1.94 ± 2.05	<0.001
	Total	289 (61%)	109 (87.2%)	398 (66.4%)	<0.001
General SE Duration	1 Day	161 (39.6%)	51 (44.3%)	212 (40.6%)	0.155
	3 Days	190 (46.7%)	41 (35.7%)	231 (44.3%)	0.137
	5 Days	22 (5.4%)	6 (5.2%)	28 (5.4%)	0.940
	1 Week	21 (5.2%)	5 (4.3%)	26 (5%)	0.834
	>1 Week	12 (2.9%)	12 (10.4%)	24 (4.6%)	0.001
	>1 Month	1 (0.2%)	0 (0%)	1 (0.2%)	1.000 *
General SE	Intensity (*μ* ± SD)	2.57 ± 2.16	4.54 ± 2.75	2.98 ± 2.43	<0.001
	Total	413 (87.1%)	115 (92%)	528 (88.1%)	0.134
Severe SE	Total	2 (0.4%)	4 (3.2%)	6 (1%)	0.019 *

Chi-squared test, Fisher’s exact test (*) and Mann–Whitney test were used with a significance level of <0.05.

**Table 5:** COVID-19 Vaccines Oral Side Effects of Participating German Healthcare Workers, February–March 2021 (*n* = 599).

Variable	Outcome	mRNA Vaccine	Viral Vector Vaccine	Total	Sig.
Oral SE	Ulcers	8 (1.7%)	4 (3.2%)	12 (2%)	0.286 *
	Vesicles	22 (4.6%)	16 (12.8%)	38 (6.3%)	0.001 *
	Blisters	1 (0.2%)	1 (0.8%)	2 (0.3%)	0.374 *
	Angular Cheilitis	2 (0.4%)	1 (0.8%)	3 (0.5%)	0.505 *
	White/Red Plaque	3 (0.6%)	4 (3.2%)	7 (1.2%)	0.038 *
	Oral Paraesthesia	11 (2.3%)	2 (1.6%)	13 (2.2%)	1.000 *
	Taste Disturbance	4 (0.8%)	8 (6.4%)	12 (2%)	0.001 *
	Xerostomia	0 (0%)	3 (2.4%)	3 (0.5%)	0.009 *
	Halitosis	9 (1.9%)	13 (10.4%)	22 (3.7%)	<0.001 *
	Bleeding Gingiva	11 (2.3%)	15 (12%)	26 (4.3%)	<0.001
	Swollen Mucosa	8 (1.7%)	5 (4%)	13 (2.2%)	0.158 *
	Total	59 (12.4%)	47 (37.6%)	106 (17.7%)	<0.001
Oral SE Onset	1–3 days	29 (37.7%)	30 (60%)	59 (46.5%)	<0.001
	1st Week	25 (32.5%)	12 (24%)	37 (29.1%)	0.074
	2nd Week	12 (15.6%)	3 (6%)	15 (11.8%)	1.000 *
	3rd Week	6 (7.8%)	3 (6%)	9 (7.1%)	0.404 *
	4th Week	5 (6.5%)	2 (4%)	7 (5.5%)	0.640 *
Ulcers/Vesicles/Blisters Location	Tongue	5 (18.5%)	7 (41.2%)	12 (27.3%)	0.164 *
	Palate	8 (29.6%)	3 (17.6%)	11 (25%)	0.486 *
	Labial/Buccal Mucosa	10 (37%)	9 (52.9%)	19 (43.2%)	0.300
	Gingiva	2 (7.4%)	1 (5.9%)	3 (6.8%)	1.000 *
	Lips	9 (33.3%)	4 (23.5%)	13 (29.5%)	0.488
White/Red Plaque Location	Tongue Dorsum	1 (33.3%)	3 (75%)	4 (57.1%)	0.486 *
	Soft Palate	1 (33.3%)	2 (50%)	3 (42.9%)	1.000 *
	Labial/Buccal Mucosa	2 (66.7%)	2 (50%)	4 (57.1%)	1.000 *

Chi-squared test and Fisher’s exact test (*) were used with a significance level of <0.05.

**Table 6:** COVID-19 Vaccines Skin-related Side Effects of Participating German Healthcare Workers, February–March 2021 (*n* = 599).

Variable	Outcome	mRNA Vaccine	Viral Vector Vaccine	Total	Sig.
Skin-related SE	Rash	12 (2.5%)	5 (4%)	17 (2.8%)	0.369
	Urticaria	2 (0.4%)	2 (1.6%)	4 (0.7%)	0.194 *
	Angioedema	2 (0.4%)	2 (1.6%)	4 (0.7%)	0.194 *
	Total	14 (3%)	7 (5.6%)	21 (3.5%)	0.171 *
Skin-related SE Location	Face	8 (57.1%)	4 (57.1%)	12 (57.1%)	1.000 *
	Upper Limb	6 (42.9%)	2 (28.6%)	8 (38.1%)	0.656 *
	Lower Limb	3 (21.4%)	1 (14.3%)	4 (19%)	1.000 *
	Trunk	0 (0%)	2 (28.6%)	2 (9.5%)	0.100 *
	Back	1 (7.1%)	1 (14.3%)	2 (9.5%)	1.000 *

Chi-squared test and Fisher’s exact test (*) were used with a significance level of <0.05.

**Table 7:** COVID-19 Vaccines Side Effects of Participating German Healthcare Workers Stratified by Gender, February–March 2021 (*n* = 596).

Variable	Outcome	mRNA-Based Vaccine			Viral Vector Vaccine		
		Female	Male	Sig.	Female	Male	Sig.
Local SE	Injection Site Pain	272 (77.9%)	93 (76.2%)	0.697	63 (75%)	23 (56.1%)	0.032
	Injection Site Swelling	61 (17.5%)	25 (20.5%)	0.458	15 (17.9%)	5 (12.2%)	0.418
	Injection Site Redness	35 (10%)	15 (12.3%)	0.484	7 (8.3%)	4 (9.8%)	0.792
	Intensity (*μ* ± SD)	1.05 ± 0.80	1.09 ± 0.83	0.715	1.01 ± 0.75	0.78 ± 0.91	0.033
	Total	273 (78.2%)	95 (77.9%)	0.935	65 (77.4%)	23 (56.1%)	0.014
Systemic SE	Fever	37 (10.6%)	9 (7.4%)	0.302	45 (53.6%)	15 (36.6%)	0.074
	Chills	52 (14.9%)	14 (11.5%)	0.348	51 (60.7%)	21 (51.2%)	0.313
	Headache/Fatigue	179 (51.3%)	47 (38.5%)	0.015	68 (81%)	25 (61%)	0.016
	Muscle Pain	99 (28.4%)	33 (27%)	0.780	47 (56%)	19 (46.3%)	0.312
	Joint Pain	55 (15.8%)	12 (9.8%)	0.107	44 (52.4%)	15 (36.6%)	0.097
	Nausea	28 (8%)	10 (8.2%)	0.952	19 (22.6%)	7 (17.1%)	0.473
	Malaise	64 (18.3%)	24 (19.7%)	0.745	43 (51.2%)	18 (43.9%)	0.444
	Lymphadenopathy	36 (10.3%)	6 (4.9%)	0.072	7 (8.3%)	6 (14.6%)	0.351 *
	Intensity (*μ* ± SD)	1.58 ± 1.81	1.27 ± 1.56	0.142	3.86 ± 2.14	3.07 ± 2.41	0.079
	Total	219 (62.8%)	68 (55.7%)	0.172	77 (91.7%)	32 (78%)	0.032
Severe SE	Total	2 (0.6%)	0 (0%)	1.000 *	3 (3.6%)	1 (2.4%)	1.000 *
Oral SE	Total	41 (11.7%)	18 (14.8%)	0.388	31 (36.9%)	16 (39%)	0.818
Skin-related SE	Total	13 (3.7%)	0 (0%)	0.129	5 (6%)	2 (4.9%)	1.000 *

Chi-squared test, Fisher’s exact test (*) and Mann–Whitney test were used with a significance level of <0.05.

**Table 8:** COVID-19 Vaccines Side Effects of Participating German Healthcare Workers Stratified by Age, February–March 2021 (*n* = 599).

Variable	Outcome	mRNA-Based Vaccine			Viral Vector Vaccine		
		≤39 Years-Old	>39 Years-Old	Sig.	≤39 Years-Old	>39 Years-Old	Sig.
Local SE	Injection Site Pain	186 (77.8%)	181 (77%)	0.834	45 (71.4%)	41 (66.1%)	0.523
	Injection Site Swelling	45 (18.8%)	43 (18.3%)	0.882	11 (17.5%)	9 (14.5%)	0.653
	Injection Site Redness	30 (12.6%)	21 (8.9%)	0.204	5 (7.9%)	6 (9.7%)	0.731
	Intensity (*μ* ± SD)	1.09 ± 0.83	1.04 ± 0.78	0.649	0.97 ± 0.78	0.90 ± 0.84	0.473
	Total	188 (78.7%)	183 (77.9%)	0.835	46 (73%)	42 (67.7%)	0.518
Systemic SE	Fever	24 (10%)	23 (9.8%)	0.926	27 (42.9%)	33 (53.2%)	0.246
	Chills	35 (14.6%)	31 (13.2%)	0.648	33 (52.4%)	39 (62.9%)	0.234
	Headache/Fatigue	133 (55.6%)	95 (40.4%)	0.001	45 (71.4%)	48 (77.4%)	0.443
	Muscle Pain	69 (28.9%)	64 (27.2%)	0.692	33 (52.4%)	33 (53.2%)	0.925
	Joint Pain	45 (18.8%)	23 (9.8%)	0.005	27 (42.9%)	32 (51.6%)	0.327
	Nausea	22 (9.2%)	17 (7.2%)	0.435	15 (23.8%)	11 (17.7%)	0.403
	Malaise	48 (20.1%)	41 (17.4%)	0.462	31 (49.2%)	30 (48.4%)	0.927
	Lymphadenopathy	27 (11.3%)	16 (6.8%)	0.089	9 (14.3%)	4 (6.5%)	0.151
	Intensity (*μ* ± SD)	1.69 ± 1.80	1.32 ± 1.69	0.006	3.49 ± 2.36	3.71 ± 2.15	0.592
	Total	161 (67.4%)	128 (54.5%)	0.004	53 (84.1%)	56 (90.3%)	0.300
Severe SE	Total	2 (0.8%)	0 (0%)	0.499 *	1 (1.6%)	3 (4.8%)	0.365 *
Oral SE	Total	33 (13.8%)	26 (11.1%)	0.366	26 (41.3%)	21 (33.9%)	0.393
Skin-related SE	Total	7 (2.9%)	7 (3%)	0.974	2 (3.2%)	5 (8.1%)	0.273 *

Chi-squared test, Fisher’s exact test (*) and Mann–Whitney test were used with a significance level of <0.05.

**Table 9:** Risk Factors of COVID-19 Side Effects of German Healthcare Workers, February–March 2021 (*n* = 599).

Predictor		mRNA-Based Vaccine		Viral Vector Vaccine		Total SE	
		OR (CI 95%)	Sig.	OR (CI 95%)	Sig.	OR (CI 95%)	Sig.
Gender	Female (vs. Male)	0.833 (0.441–1.573)	0.573	3.429 (0.910–12.912)	0.069	1.048 (0.603–1.819)	0.869
Age Group	≤ 39 years (vs. > 39 years)	1.141 (0.666–1.955)	0.630	1.018 (0.279–3.705)	0.979	1.122 (0.683–1.842)	0.650
Infection	No (vs. Yes)	21.310 (2.180–208.303)	0.009			7.721 (1.528–39.017)	0.013
Chronic Illness	No (vs. Yes)	0.911 (0.501–1.657)	0.760	2.173 (0.571–8.270)	0.255	1.058 (0.615–1.823)	0.838
Medical Treatment	No (vs. Yes)	1.032 (0.589–1.806)	0.913	3.600 (0.965–13.428)	0.057	1.281 (0.767–2.139)	0.344

**Table 10:** Risk Factors of mRNA-based COVID-19 Vaccine Side Effects of German Healthcare Workers, February–March 2021 (*n* = 474).

Predictor		Local SE		Systemic SE		Total SE	
		OR (CI 95%)	Sig.	OR (CI 95%)	Sig.	OR (CI 95%)	Sig.
Gender	Female (vs. Male)	1.021 (0.621–1.679)	0.935	1.338 (0.881–2.032)	0.172	0.833 (0.441–1.573)	0.573
Age Group	≤ 39 years (vs. > 39 years)	1.047 (0.677–1.621)	0.835	1.725 (1.188–2.505)	0.004	1.141 (0.666–1.955)	0.630
Infection	No (vs. Yes)	11.100 (1.142–107.864)	0.038	4.747 (0.490–45.986)	0.179	21.310 (2.180–208.303)	0.009
Chronic Illness	No (vs. Yes)	0.863 (0.530–1.406)	0.555	1.105 (0.739–1.654)	0.626	0.911 (0.501–1.657)	0.760
Medical Treatment	No (vs. Yes)	1.084 (0.689–1.706)	0.728	0.905 (0.614–1.333)	0.613	1.032 (0.589–1.806)	0.913

**Table 11:** Risk Factors of Viral Vector-based COVID-19 Vaccine Side Effects of German Healthcare Workers, February–March 2021 (*n* = 125).

Predictor		Local SE		Systemic SE		Total SE	
		OR (CI 95%)	Sig.	OR (CI 95%)	Sig.	OR (CI 95%)	Sig.
Gender	Female (vs. Male)	2.677 (1.202–5.965)	0.016	3.094 (1.061–9.022)	0.039	3.429 (0.910–12.912)	0.069
Age Group	≤39 years (vs. >39 years)	1.289 (0.597–2.783)	0.519	0.568 (0.193–1.671)	0.304	1.018 (0.279–3.705)	0.979
Chronic Illness	No (vs. Yes)	1.182 (0.492–2.836)	0.709	2.016 (0.667–6.094)	0.214	2.173 (0.571–8.270)	0.255
Medical Treatment	No (vs. Yes)	2.739 (1.162–6.455)	0.021	2.125 (0.701–6.441)	0.183	3.600 (0.965–13.428)	0.057

## Data Availability

The data that support the findings of this study are available from the corresponding author upon reasonable request.
